# Impacts of the invasive species *Caulerpa cylindracea* Sonder, 1845 on the algae flora of the west coast of Algeria

**DOI:** 10.3897/BDJ.9.e64535

**Published:** 2021-05-26

**Authors:** Benabdallah Bachir Bouiadjra, Malika Ghellai, Mohamed Daoudi, Ibrahim Elkhalil Behmene, Mohammed El Amine Bachir Bouiadjra

**Affiliations:** 1 Laboratory of Animal Production Sciences and Techniques (LSTPA), University of Mostaganem, Mostaganem, Algeria Laboratory of Animal Production Sciences and Techniques (LSTPA), University of Mostaganem Mostaganem Algeria; 2 University of Relizane, Relizane, Algeria University of Relizane Relizane Algeria

**Keywords:** algae, invasive, macrophytes, diversity, index, phytosociological, taxon

## Abstract

The assessment of the impacts of the expansion of the invasive species on taxonomic diversity, the abundance and dominance of groups of algae, the presence and/or absence of species of ecological interest that may or may not be indicative of water quality well mentioned, through the installation of a 20 × 20 cm quadrat representing the minimum area. The observation stations were visited monthly, during a repetitive three-year cycle, during the spring, summer and autumn seasons, periods of maximum growth and development of the algal flora and the results suggest the following facts. The invasive alga *Caulerpa cylindracea* Sonder, 1845 tends to colonise disturbed ecosystems reflecting a reduction in native algal diversity; in fact, we note a drastic impoverishment of the invaded algal community, represented by a limited number of Macrophyte algae accompanying the invasive taxon in phytosociological surveys and a Shannon-Weaver Diversity Index (H’) and Equitability reduced by 4.49 and 0.77 n the heavily affected station. The number of macroalgal species accompanying the invasive species has dropped by 52% in Salamandre. In addition, the multidimensional analysis, represented by the Hierarchical Ascendant Clustering applied to this case, confirms our results.

## Introduction

Studies on ENI date back to the 1970s (Ojaveer et al. 2018). Since then, global research in this area has developed very rapidly (Giakoumi et al. 2019).

The Mediterranean Sea is today considered to be one of the most affected of the regional seas, due to the various anthropogenic pressures on the different ecosystems of the Mediterranean asin (Mannino et al. 2017). This favors it to be an important place for non-native species, which can become invasive leading to a loss of biodiversity and changes in ecosystem services (Brunel et al. 2013, Giakoumi 2014).

The presence of mud-flats, estuaries and coral reefs along lagoons and rocky beaches provides ideal habitat for sustainable algae growth (Premarathna et al. 2019).

The concept of invasive species introduction is applied when the naturalisation of a species outside its usual geographic range is directly or indirectly linked to a human activity (Carlton 1985). The impact of introduced species has, thus, become the second most important cause of biodiversity loss in the world (Wilson 1997), after the outright disappearance of habitats. Bright (1998) defines the impact of introduced species on the planet's biodiversity as "evolution in reverse".

Amongst these bioinvasions, we are interested in *Caulerpa cylindracea* (Sonder 1845), recently reported on the Algerian west coast and which presents a strongly invasive behaviour during its colonisation (Manconi et al. 2020). It is considered to be one of the most threatening invasions of the Mediterranean Sea (Piazzi et al. 2016) and its impacts on algal biodiversity and seagrass beds are well reported in literature (Gribben et al. 2018, Montefalcone et al. 2015).

**Figure 1.**
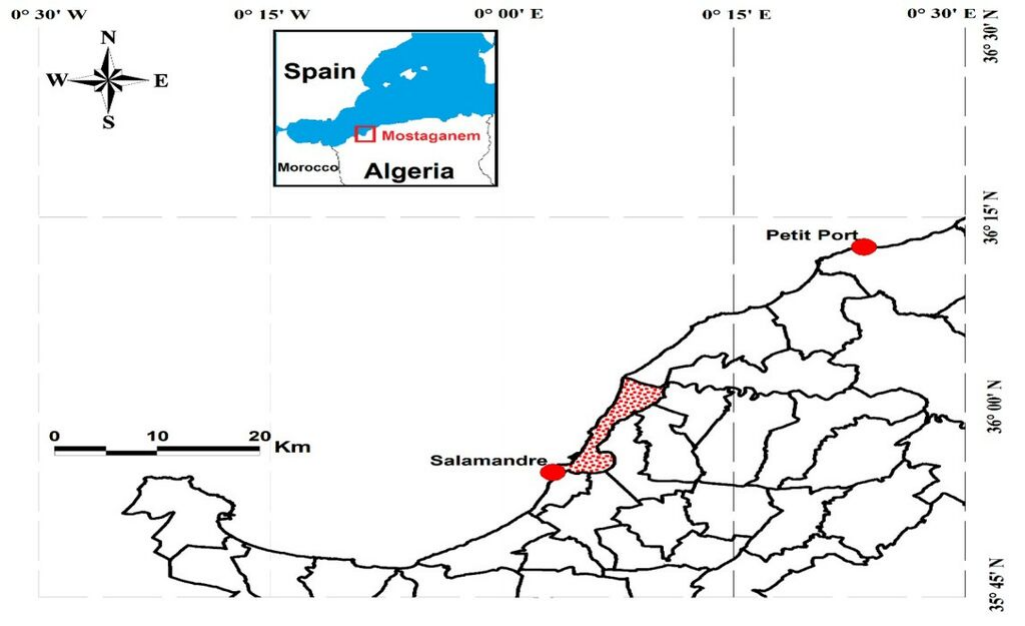
The geographical location of the studied sites (Google Earth modified).

Indeed, the algal flora of the Algerian west coast remains largely unexplored. Direct observation of the algal settlement constituting the phytobenthos and its evolution is essential for monitoring possible changes in algal biodiversity. Therefore, the purpose of this research is to:

- establish a quantitative and qualitative inventory, located in time and space, of macrophyte species inhabiting two sites located on the coast of Mostaganem (west coast of Algeria Fig. 1), one affected by *C. cylindracea*, the other spare.
- assess the impact of invasive species expansion on taxonomic and specific diversity, abundance and dominance of algal groups, presence and/or absence of species of ecological interest that may or may not be indicative of water quality.

## Material and methods

The choice of harvesting stations was primarily based on the importance of the algal marine flora, the location of the stations studied shown in Fig. 1.

The area studied extends over 50 km of coastline, from Salamandre each to Sidi Lakhdar each (small port). Both stations were visited monthly during a repetitive cycle of three years, in spring, summer and autumn seasons, periods of maximum growth and development of the algal flora. In each station, we prospected a 50 to 100 m long and 10 to 15 m wide coastal line, with a depth of 5 to 10 m.

**Salamandre Station**: The Salamandre site (Fig. 1) is 5 km from the town of Mostaganem, housing a new fishing port, its length around 4000 m. Surveys conducted in sheltered, quiet areas within a rocky beach with the following coordinates: 35°55' N/ 0°03' E.

**Small Port Station**: This zone is located 35 km east of Mostaganem (Fig. 1), the sampling stations located in places with sandy and rocky bottoms on a beach 3500 m long sheltering a clear-seeded *Posidonia oceanica* meadow, associated with macrophyte algae with geographical coordinates, 36°12' N / 0°23' E.

**Sampling, sorting and conservation of material.**

**Figure 2.**
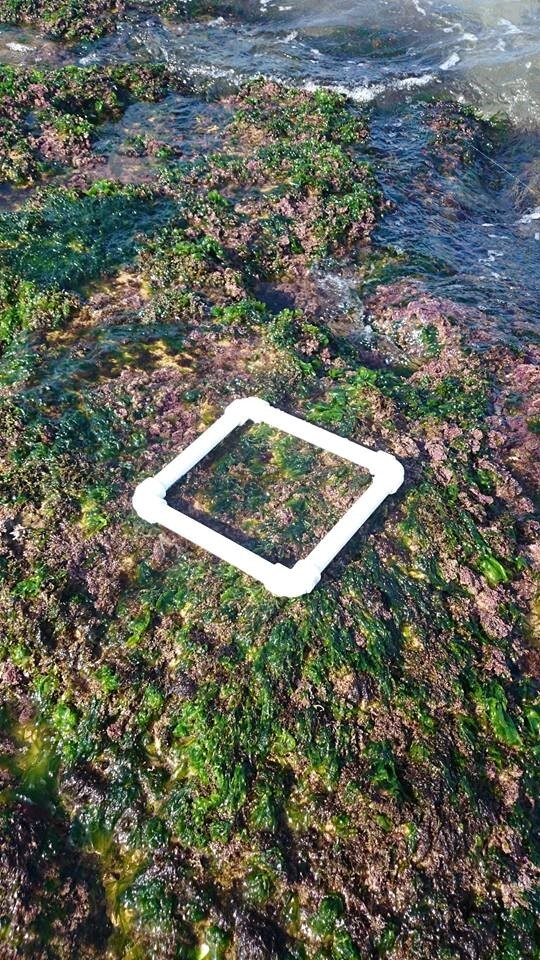
Quadrat used for sampling (20 × 20 cm).

Sampling was performed on both soft and hard variable substrates. Sampling random, raking the rocks at each survey, three surveys per observation station and per year, according to climatological conditions and without biotope delimitation so that the sample is as representative as possible of the zone (Fig. 2).

**Species identification**

Different keys for the determination of macrophyte algal flora have been consulted see Fischer et al. 1987, Mojetta and Ghisotti 1996, Gayral 1958, Gayral 1966, ‎Carrillo Marta Sanson 1999, Coppejans 1983, Hamel 1931, Hamel 1939,Verlaque 1990, Ballesteros et al. 2007, Boudouresque and Boudouresque 1969, Feldmann 1931, Feldmann 1933, Bliding 1968, Belhissoun 1995, allow us to draw up a catalogue which is by no means exhaustive of the species of algae inhabiting the two stations considered.

**Minimum Area**

The minimum area is the area where one has the best chance of finding all the species of the settlementstudied to within 10% according to Gounout (1969). For our case, the minimum area adopted is 20 × 20 cm a surface of 400 cm^2^ Fig. 2.

**Analytical Parameters**

For the quantitative and qualitative analysis of vegetation, we used the terrestrial phytosociological methods that several authors have adopted in the marine environment (Boudouresque 1971a, Boudouresque 1971b, Cormaci et al. 1997, Scammacca et al. 1993). The results are expressed by their average, with data analysis carried out by the Statistica software (version 7.0) for all the parameters retained.

**Recovery**

The recovery (**Ri**) is the approximate percentage of the substrate surface covered in projection by species **i**. Given the stratification of vegetation, the total coverage of a survey ∑**Ri** is generally greater than 100%. The importance of recovery is expressed in class according to the following scale:

+ = negligible recovery

**1** = less than 5% of the surface is covered

**2** = between 5 and 25% of the surface area

**3** = between 25 and 50% of the surface area

**4** = between 50% and 75% of the surface area

**5** = more than 75% of the surface area

**Overall average recovery**

Each class of the recovery coefficient Ri is assigned a conventional monthly average value (class centre) called average recovery.

Absence = 0; + = 01%; 1 = 2.5%; 2 = 15%; 3 = 37.5%; 4 = 62.5%; 5 = 87.5%

The RMG (overall average recovery) of species i in a set of N recorded is the average of these successive average recoveries.

**RMG = ∑Ri/N**

The RMG of a subset E of n species (ecological group, systematic unit or phytogeographic elements) is the sum of the constituent species.

**Species richness (Q).**

The Q coefficient is the specific population size of any subset in a survey (ecological group, systematic unit or biogeographic features) and the Q (highlighted) of a group of species representing its average species size.

**Quantitative dominance.**

The dominance based on recovery (∑**DRi**) of a group of species in a survey (in a survey table), is the ratio, expressed as a percentage of the sum of their recovery (or their RMG) to the total recovery of species in the survey (or to the total average recovery of species in the survey table).

**Qualitative dominance (DQ̄)**

This is the ratio expressed as a percentage of Q, (where Q highlighted) to T (where T highlighted).

**Index of Specific Diversity** (Shannon-Wiener)

This ndex (H') measures, in a settlement, the amount of information resulting from species differentiation. The H' value reflects the degree of structural evolution, maturity and stability of the ecosystem under consideration. It was calculated from the dominance of each species (Ri/Rt) according to Shannon's formula (in Boudouresque and Cinelli 1971).

H' = - ∑Pi * Log_2_ Pi with **Pi = Ri/Rt Rt** = total recovery

he calculations were made from Suppl. materials 1, 2 under R software version 3.0.3 R Core Team 2014

**Evenness or Regularity E**

This is the ratio between the actual community diversity index and its maximum value for the number of species present **H'_max_ = Log_2_ T** with **T** = total number of taxa

**E = H'/H'_max_**

the calculations were made from Suppl. materials 1, 2 under R software version 3.0.3 R Core Team 2014

**Taxonomic diversity**

Three taxonomic indices have also been adopted for the study of taxonomic diversity (∆, ∆* and ∆+). These were calculated from a taxonomic tree constructed on the basis of phylogenetic classification (Clarke and Warwick 1998), considering eight taxonomic levels: species, genus, family, order, class, phylum, under-reign and reign. From this tree, a taxonomic distance ωij is quantified between each pair of species, assuming that this distance is equal to 100 for two species related to the highest taxonomic level considered in the study (Clarke and Warwick 1999). This calculated distance (ωij) will then be integrated into the calculation of the various taxonomic indices. The calculations were performed using Suppl. materials 3, 1, 2 software R version 3.0.3 (R Core Team 2014). The phylogenetic classification is based on the Worms Editorial Board (Guiry and Guiry 2020).

With xi (i = 1, ..., S): the abundance of the i^th^ species, N (= Σ xi): the total number of individuals in the sample and ωij: the distance to be covered between species i and the first common node with species j in the hierarchical classification:

- ∆"**Taxonomic diversity**" (Warwick and Clarke 1995):

\begin{varwidth}{50in}
        \begin{equation*}
            ‎∆ =2*{∑∑(i<j)ωij xi xj \over N(N-1)}
        \end{equation*}
    \end{varwidth}

∆ represents the average taxonomic distance between each pair of randomly selected individuals in the sample.

- ∆*"**Taxonomic Distinctness**" (Warwick and Clarke 1995):

\begin{varwidth}{50in}
        \begin{equation*}
            ‎∆^* ={∑∑(i<j)ωij xi xj \over ∑∑(i<j)xi xj}
        \end{equation*}
    \end{varwidth}

∆* is the average taxonomic distance between two randomly selected individuals belonging only to different species.

- ∆+"**Average Taxonomic Distinctness**", on data in presence/absence, Δ and Δ* are simplified by Δ+ (Clarke and Warwick 1998):

\begin{varwidth}{50in}
        \begin{equation*}
            Δ^+=2*{∑∑ (i<j) ωij \over S(S-1))}
        \end{equation*}
    \end{varwidth}

∆+ measures the average taxonomic distance between two randomly selected species.

- **Funnel Test**:

In order to detect assemblages whose taxonomic diversity would be influenced by disturbances, Warwick and Clarke (1998) proposed the "Funnel" test. This test consists comparing the taxonomic variety of a site to the area to which it belongs, from 1000 random draws of size m species made on the global list of S species listed in the area under consideration.

**Hierarchical Ascendant Clustering (HAC)**

The hierarchical classification has been used in a wide variety of disciplines. It has been described by several authors including Daget 1979, Dagnelie 1986, Legendre and Legendre 1984, Legendre and Legendre 1998, Herrera and Legac 2002 and Bouroche and Saporta 2005. Our study was confirmed by the use of this multidimensional analysis which consists in partitioning the objects (or descriptors) of the study into groups and subgroups, passing through the condensation of the information provided through the data matrix into a similarity or distance matrix. The latter will then be classified according to different classification algorithms (Ward, single, complete, average), which will each provide a corresponding dendrogram. The calculation procedure for this analysis was performed using Suppl. materials 1, 2 under software R version 3.0.3 (R Core Team 2014).

## Results

**Figure 3.**
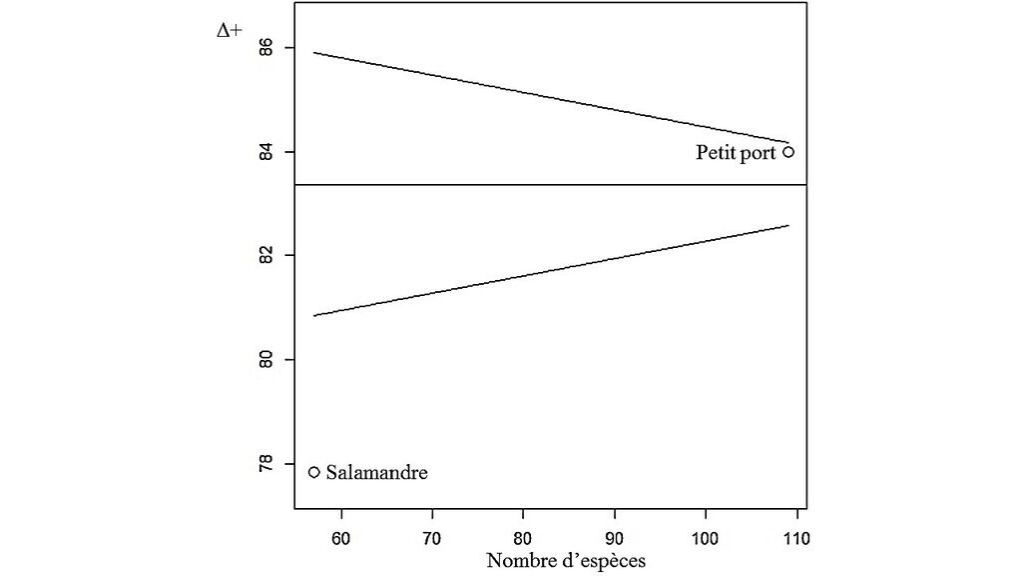
Funnel test.

**Figure 4.**
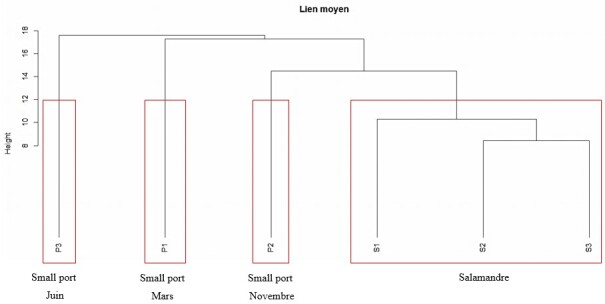
Classification of phytosociological surveys carried out at the Small Port and the Salamandre Station by the average link.

Results are reported in Table 1 and Table 3 for algal stand composition at the Salamandre and Small Port Stations respectively and in Tables 2, 4 for analytical parameters (RMG, DRi, Q̄ DQ̄) at the same stations. Table 5 represents the set of taxonomic indices at the two study stations and Fig. 3 illustrates the Funnel Test. The Hierarchical Ascendant Classification of the study stations based on the algae stands surveyed is illustrated in Fig. 4.

The results shown in Table 1 and Table 3, with illustrations and descriptions of sample specimens for the two observation stations are contained in a doctoral thesis (‎Bachir Bouiadjra 2012).

## Discussion

### Salamandre Station

**Figure 5.**
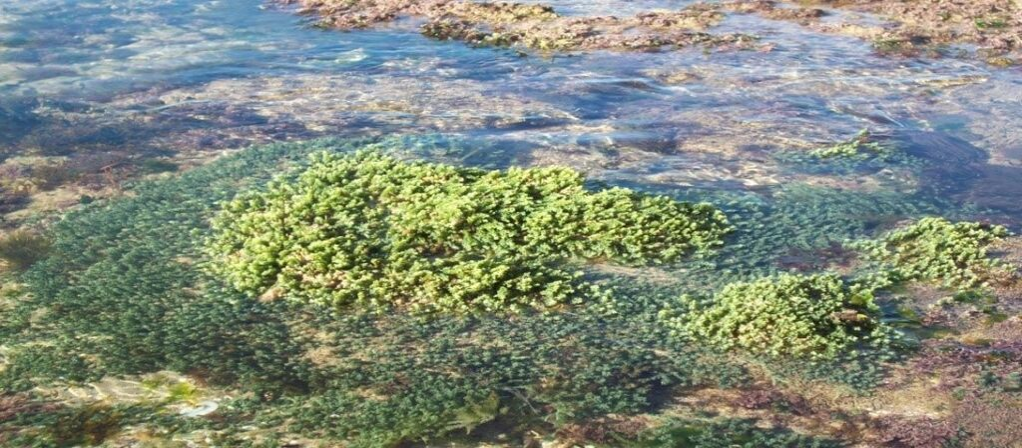
Central colonies of *C. cylindracea* at the Salamandre Station.

**Figure 6.**
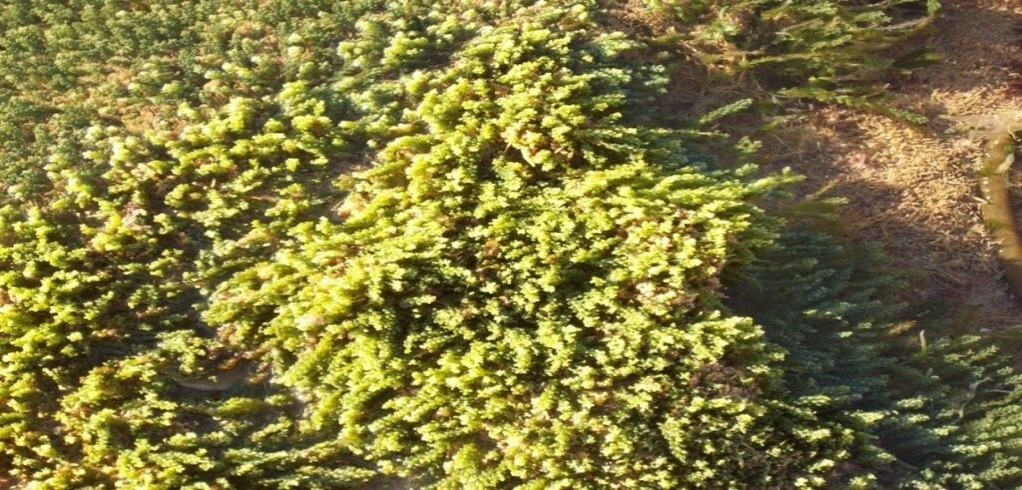
Dense colonies of *Caulerpa cylindracea* in the heavily affected station of Salamandre.

The reading of the sheet on the algal settlement of the Salamandre site (Table 1) and the result of the structural parameters shown in Table 2, indicate a quantitative and qualitative dominance, respectively of 52% and 46% with an overall average recovery exceeding 50% of the species belonging to Chlorophyceae. Within this systematic unit, we note an overall average recovery rate of five invasive taxa, *C. cylindracea*, *C. fragile*, *A. armata*, *F. rufolanosaL. lallemandii*, which is of the order of 24.5%, 10.5% of which is reserved for the *C. cylindracea*, which is progressing in some surveys by joining its small colonies (Figs 5, 6) to which are added the eutrophication indicator species of the environment and nitrophilic strongly accumulating nitrogen are represented by the order of Ulvales in the number of 8 species (Table 1) with a global average cover rate of 18% on a total global cover of 50% of Chlorophyceae in the Salamandre Station. This high presence rate is an indicator of a polluted environment which is confirmed by domestic and urban discharges (Khiari et al. 2017, Damak et al. 2019) duly observed at Salamandre Station, in addition the presence rate of the invasive species *C. cylindracea* amount to 10.83% (Table 1) which confirms an increasingly advanced colonisation.

The algal population of Phaeophyceae occupies a negligible average cover rate of 2.85% at the Salamandre Station and reveals the total absence of macrophyte species sensitive to pollution by detergents and water turbidity such as cystoseires (*Cystoseira stricta, C. algeriensis, C. crinita* ... etc) (Gamulin-Brida et al. 1967, Bellan-Santini 1966, Verlaque and Fritayre 1994a) which confirms that this site is strongly impacted by *C. cylindracea* and disturbed, given the strong presence of the invasive species On the other hand the site of the small harbor displays a rate of recovery of Phaeophyceae macrophytes of 42.61% (Table 4) with a presence of nine species of Cystoseires suggesting a good water quality and an average algal specific richness of 76 species (Table 3) per survey contrary to the previous site, which displays an average number of species of only 43 per survey (Table 1).

As for Rhodophyceae the overall cover rate is respectively 45.11% and 56.10% at the Salamandre and small port Station with a qualitative dominance of 44% and 43% (Tables 2, 4) which suggests protection and valorisation of some taxa rich in mineral elements and chemical components (Sambusiti et al. 2015, Cardoso et al. 2014, Shobana et al. 2017) see *Ellisolandia elongata*, *Hypnea musciformis*, *Gelidium crinale*, *Gelidium spinosum*, *Gelidium corneum*, *Jania longifurca*, *Jania rubens* and *Gracilaria bursa-pasoris*.

This is easily verified in the number of species per phytosociological survey, which is much lower than those reported in the Small Port site and the reduction in the Diversity and Evenness Indices, respectively by 4.49 and 0.77 at the Salamandre Station, in relation to the results obtained at the Small Port. In this station invaded by *C. cylindracea*, a native species, *Caulerpa prolifera* (Forsskal) Lamouroux are recorded in surveys, with a low recovery rate of 2.5% compared to that of the invasive taxon (10.5%) suggesting a tendency for *C. prolifera* to be replaced by the invasive species (Piazzi and Ceccherelli 2006). Over time, according to Ceccherelli et al. 2001, Jaubert et al. 2003), the homogenisation of the underwater landscape observed in some Mediterranean regions heavily affected by the invasive species has resulted. It is important to remember that for the four other invasive species of Rhodhophycea inventoried and subservient to the photophilic infralittoral of hard substrates, seem to find favorable conditions for their development, without any threat to the ecosystem. Nevertheless, the *Bonnemaisonncea* of the genus *Asparagpsis* found in Salamandre, has major assets its avoidance by herbivorous fish because secreting secondary metabolites and its ability to multiply vegetatively and by apomeiotic spores (Feldmann 1954) hence its expansion to the site of Salamandre with a recovery rate of 1%, thus forming a settlement in *Asparagpsis*.

In addition, the beach of Salamandre with coastal developments operating in recent years is a vulnerable site to closely monitor to avoid erosion of its algal richness due to an expansion of invasive taxon *C. cylindracea*.

### Small Port Station

It is a site rich in species, 109 taxa listed (Table 3), 80% of the total number recorded on the coast of Mostaganem (‎Bachir Bouiadjra 2012). The recovery rate of the entire algal flora exceeds 100%, with a Diversity Index (H') of 5.56 which is higher than that of the previous station (Salamandre), as well as that of Bordj El kifan (1.62) east of Algiers reported by Serridi (2003). The Evenness Index is about 0.82, which suggests, according to Daget (1979), a balanced algal stand.

The overall average overlap among Rhodophyceae is clearly dominant at 56%, the same observation is made for their quantitative and qualitative dominance estimated at 43%, followed by the Phaeophyceae group with an RMG of 42% and dominance (Dri) and (DQ) of 32% and finally the Chlorophyceae group with an RMG of 31% (Table 4). This represents a well-structured settlement with species constituting the crusty, grassy, erect and epiphytic strata according to the algal settlement sheet (Table 3). Note the absence of the invasive taxon *C. cylindracea* however, the fishing port adjacent to the observation sites, will constitute a potential vector for caulerpe transmission in the area. Hence the need for increased vigilance and follow-up with awareness campaigns among fishing professionals to avoid contamination of the site.

### Taxonomic Diversity Indices

Analysis of the different taxonomic Diversity Indices (Table 5) indicates a higher taxonomic diversity at the Small Port than at the Salamandre Station. This means that the species recorded at the Small Port are more taxonomically distant than those found in Salamandre. In other words, the average taxonomic distance between each pair of randomly selected individuals is greater at the Small Port than at Salamandre.

The Funnel Test (Fig. 3) based on the values of ∆+ obtained for each Station, in comparison with the mean expected value in the study area, shows that the Salamandre Station is outside the 95% confidence interval of ∆+m, indicating a low taxonomic diversity compared to that expected in the area. This suggests that this site is subject to anthropogenic disturbances, influencing the taxonomic composition of the settlement by generating a poor assemblage of species, in addition to the presence of invasive species in this plant, associated with untreated wastewater discharges, confirm this reduction in algal diversity (Verlaque and Fritayre 1994b, Ceccherelli et al. 2001, Piazzi et al. 2001, Piazzi and Cinelli 2003, Piazzi and Ceccherelli 2006).

### Hierarchical Ascendant Clustering

Our study was confirmed by a multidimensional analysis which is the Hierarchical Ascendant Clustering (HAC). The latter (Fig. 4) shows a clear separation between the two sites considered by indicating four distinct groups of phytosociological surveys. The first two, represented by the summer and spring surveys at the Small Port Station, are the most dissimilar of the other groups and represent the greatest distance. The two surveys, therefore, represent the most species-rich removal recorded, indicating 89 and 72 species recorded respectively during the spring and summer seasons at the Small Port. However, we note a decrease in the number of species in the surveys conducted during the winter season at the same site with 68 species recorded, which results in a smaller distance in the classification tree for this survey. The last group consists of the surveys carried out in the Salamandre Station and represents the smallest distance in the dendrogram due to the low number of species recorded due to the dominance of the settlementby species belonging to Chlorophytes, neutrophils associated with five other invasive species including *C. cylindracea*, *C. fragile*, *A. armata*, *F. rufolanosa* and *L. lallemandii*, which testifies to the disturbance and degradation of the environment in the Salamandre Station.

## Conclusions

The invasive alga *Caulerpa cylindracea* Sonder, 1845 tends to colonise disturbed ecosystemseflecting a reduction in indigenous algal diversity (Ceccherelli et al. 2001, Piazzi et al. 2001, Piazzi and Cinelli 2003, Piazzi and Ceccherelli 2006). This is confirmed in the heavily colonised observation site of Salamandre Beach, through the statistical analysis tools used to see the parameters of abundance recovery, qualitative and quantitative dominance of algal groups, Equitability Index and taxonomic diversity and where there is a drastic depletion of the invaded algal community, represented by a limited number of Macrophyte algae accompanying the invasive taxon in phytosociological surveys and a low Shannon-Weaver Diversity Index (H) of 4.49. On the other hand, the Station of the Small Port not affected by *C. cylindracea* hosts a population exceeding in some surveys about twenty taxa or the Diversity Index is 5.56 suggesting a well-structured and stable algal population. The number of macroalgal species accompanying the invasive species has dropped by 52% in Salamandre. Verlaque and Fritayre (1994b) attribute this decrease to a rate ranging from 25 to 55%. This is consistent with our results. Furthermore, the analysis of the floral procession mentioned in the surveys of the Salamandre Station indicates a decrease in the cover of erect shrub algae (-70 to -80%) and epiphytic and filamentous algae in the autumn season, while encrusting algae of the genus *Corallina* dominate and best resist the occupation of the ecological niche by the invasive species *C. cylindracea*. This study provides an inventory of the algal population associated with the recentlyintroduced *C. cylindracea* at the Mostaganem coast, which needs to be monitored at different levels of the affected coastline in order to effectively limit its expansion to the detriment of existing algal diversity.

## Supplementary Material

F231F485-78EF-5FEF-8497-AAB7D1090FB2 10.3897/BDJ.9.e64535.suppl1 Supplementary material 1 average recovery: Salamandre Station Data type Table.CSV Brief description average recovery by species and by season, Salamandre Station File: oo_538671.csv https://binary.pensoft.net/file/538671 Bachir Bouiadjra B., Ghellai M., Daoudi M., Behmene I.E., Bachir Bouiadjra M.A.

C1C1B0FA-A154-5302-BE8C-C3E7EE4A0F7E 10.3897/BDJ.9.e64535.suppl2 Supplementary material 2 average recovery: Small Port Station Data type Table.CSV Brief description average recovery by species and by season, Small Port Station File: oo_538672.csv https://binary.pensoft.net/file/538672 Bachir Bouiadjra B., Ghellai M., Daoudi M., Behmene I.E., Bachir Bouiadjra M.A.

98A47F6D-7461-5E84-BEA0-8F768A743F1F 10.3897/BDJ.9.e64535.suppl3 Supplementary material 3 taxonomic classification of the species recorded Data type table.CSV Brief description the taxonomic classification table, based on the phylogenetic classification, used for the calculation of taxonomic indices File: oo_538667.csv https://binary.pensoft.net/file/538667 Bachir Bouiadjra B., Ghellai M., Daoudi M., Behmene I.E., Bachir Bouiadjra M.A.

## Figures and Tables

**Table 1.** Algal settlement sheet: Salamandre Station.

Statement number	1	2	3	RMG	Pr
Month	Mar.	Nov.	June		
Surface in cm²	400	400	400		
Cover	100%	90%	90%		
Exposition	N	NE	NW		
*Erythrotrichia carnea*	+	+	+	0.10	3
*Acrochaetium cheminii*	0	+	+	0.07	2
*Colaconema daviesii*	+	+	+	0.10	3
*Asparagopsis armata*	2	1	1	6.67	3
*Falkenbergia rufolanosa*	1	1	1	2.50	3
*Dasya rigidula*	+	+	+	0.10	3
*Taenio mananum*	1	1	0	1.67	2
*Chondria coerulescens*	0	+	+	0.07	2
*Chondriamairei* + *C. capillaris*	+	+	+	0.10	3
*Digenea simplex*	1	1	+	1.70	3
*Halopithyin curvus*	0	1	1	1.67	2
Herposiphonia secunda f. secunda	0	0	+	0.03	1
Herposiphonia secunda f. tenella	0	+	0	0.03	1
*Lophocladia lallemandii*	+	+	0	0.07	2
*Vertebrata fruticulosa*	+	+	+	0.10	3
*Vertebrata furcellata*	0	+	+	0.07	2
*Polysiphonia spinulosa*	0	0	+	0.03	1
*Amphiroa rigida*	2	1	+	5.87	3
*Ellisolandia elongata*	2	2	2	15.00	3
*Corallina officinalis*	0	2	1	5.83	2
*Jania rubens*	+	1	1	1.70	3
*Hypnea musciformis*	1	1	1	2.50	3
*Grateloupia filicina*	0	+	+	0.07	2
*Gastroclonium clavatum*	0	+	+	0.07	2
*Dictyopteris polypodioides*	+	+	1	0.90	3
*Dictyopteris divaricata*	+	1	1	1.70	3
*Asperococcus bullosus*	0	0	+	0.03	1
*Feldmannia globifera*	0	+	+	0.07	2
*Colpomenia peregrina*	+	+	+	0.10	3
*Colpomenia sinuosa*	+	+	0	0.07	2
*Bryopsis hypnoides*	0	0	+	0.03	1
*Bryopsis muscosa*	0	+	0	0.03	1
*Bryopsis plumosa*	+	+	0	0.07	2
*Bryopsis secunda*	+	+	0	0.07	2
*Caulerpa prolifera*	1	1	1	2.50	3
*Caulerpa racemosa*	2	2	1	10.83	3
*Codium bursa*	0	1	0	0.83	1
*Codium effusum*	1	1	0	1.67	2
*Codium fragile*	2	1	+	5.87	3
*Codium tomentosum*	0	+	1	0.87	2
*Chaetomorpha aerea*	1	1	1	2.50	3
*Chaetomorpha mediterranea*	0	1	1	1.67	2
*Chaetomorpha linum*	0	1	1	1.67	2
*Cladophora coelothrix*	0	1	0	0.83	1
*Cladophora laetevirens*	1	0	0	0.83	1
*Cladophora prolifera*	1	1	1	2.50	3
*Cladophora coelothrix*	+	+	+	0.10	3
*Cladophora rupestris*	0	0	1	0.83	1
*Bldingia marginata*	+	+	+	0.10	3
*Ulva compressa*	1	1	1	2.50	3
*Ulva intestinalis*	2	1	1	6.67	3
*Ulva linza*	+	+	+	0.10	3
*Ulva prolifera*	+	+	+	0.10	3
*Ulva clathrata*	0	0	+	0.03	1
*Ulva fasciata*	+	+	+	0.10	3
*Ulva lactuca*	1	1	1	2.50	3
*Ulva rigida*	2	1	1	6.67	3
Number of species per statement	34	49	46	101.79	
Average number of species per statement	43			Pr: the presence of the species	
R/P per statement	3.5	4.4	4.2		
R/P average	4.03				
Diversity ndex H’	4.49				
Evenness E	0.77				

**Table 2.** Analytical parameters at the Salamandre Station.

	∑RMG	Dri	Q̄	DQ̄
Chlorophyceae	53.91%	52.96%	19.66	46.08%
Phaeophyceae	2.85%	2.79%	4	9.37%
Rhodophyceae	45.11%	44.31%	19	44.53%
Total	101.87%			

**Table 3.** Algal settlement sheet: Small Port Station.

Statement number	1	2	3	RMG	Pr		Statement number		1	2	3	RMG	Pr
Month	Mar.	Nov.	June				Date		Mar.	Nov.	June		
Surface in cm²	400	400	400				Surface in cm²		400	400	400		
Cover	100%	90%	90%				Cover		100%	90%	90%		
Exposition	N	NE	NW				Exposition		N	NE	NW		
*Porphyra umbilicalis*	+	0	0	0.03	1		*Cladosiphon mediterraneu*		+	0	0	0.03	1
*Pyropia leucosticta*	+	0	+	0.07	2		*Myriactula gracilariae*		0	0	+	0.03	1
*Sahlingia subintegra*	+	+	0	0.07	2		*Myriactula rigida*		0	0	+	0.03	1
*Antithamnion amphigeneum* A. J. K. Millar	0	0	+	0.03	1		*Myriactula rivulariae*		0	0	+	0.03	1
*Asparagopsis armata*	2	1	0	5.83	2		Ectocarpusfas ciculatus var. fasciculatus		+	0	+	0.07	2
*Falkenbergia rufolanosa*	1	1	0	1.67	2		*Ectocarpus commensalis*		0	0	+	0.03	1
*Anotrichium tenue*	+	+	0	0.07	2		*Ectocarpus siliculosus*		+	0	0	0.03	1
*Centrocera clavulatum*	+	0	+	0.07	2		*Feldmannia globifera*		+	+	0	0.07	2
*Corallophila cinnabarina*	+	+	+	0.10	3		*Feldmannia simplex*		+	+	+	0.10	3
*Ceramium diaphanum*	2	1	+	5.87	3		*Feldmannia mitchelliae*		1	+	0	0.87	2
*Spyridia filamentosa*	0	+	+	0.07	2		*Hincksia sandriana*		1	+	0	0.87	2
*Dasya rigidula*	+	+	0	0.07	2		*Ralfsia verrucosa*		1	1	0	1.67	2
*Taenio mananum*	+	+	0	0.07	2		*Cystoseira algeriensis*		1	+	1	1.70	3
*Chondria coerulescens*	+	+	+	0.10	3		*Cystoseira barbata*		1	+	+	0.90	3
*Chondria dasyphylla*	+	+	0	0.07	2		*Cystoseira compressa*		1	+	1	1.70	3
*Chondria mairei*	+	+	+	0.10	3		Cystoseira brachycarpa var. balearica		1	1	1	2.50	3
*Digenea simplex*	1	1	0	1.67	2		*Cystoseira crinita*		0	0	2	5.00	1
*Halopithys incurva*	1	1	1	2.50	3		*Cystoseira sedoides*		1	1	1	2.50	3
Herposiphonia secunda f. secunda	+	0	+	0.07	2		*Cystoseira mediterranea*		1	+	1	1.70	3
Herposiphonia secunda f. tenella	+	0	+	0.07	2		Cystoseira amentacea var. stricta		1	+	1	1.70	3
*Laurencia microcladia*	1	+	1	1.70	3		*Cystoseira tamariscifolia*		1	1	1	2.50	3
*Laurencia obtusa*	0	1	1	1.67	2		*Sargassum acinarium*		0	0	1	0.83	1
*Palisada perforata*	+	1	1	1.70	3		*Sargassum vulgare*		1	0	+	0.87	2
*Lophocladia lallemandii*	+	0	0	0.03	1		*Colpomenia peregrine*		1	1	1	2.50	3
*Ellisolandia elongata*	2	1	+	5.87	3		*Colpomenia sinuosa*		1	1	1	2.50	3
*Corallina officinalis*	1	1	1	2.50	3		*Cladostephus spongiousus*		1	+	0	0.87	2
*Jania longifurca*	1	+	+	0.90	3		*Sphacelaria cirrosa*		+	0	+	0.07	2
*Jania rubens*	1	+	+	0.90	3		*Sphacelaria plumula*		+	0	+	0.07	2
*Gelidiella ramellosa*	+	0	1	0.87	2		*Halopteris scoparia*		0	1	1	1.67	2
*Gelidium crinale*	+	0	1	0.87	2		*Bryopsis duplex*		+	+	0	0.07	2
*Gelidium spinosum*	0	0	1	0.83	1		*Bryopsis hypnoides*		+	+	0	0.07	2
*Gelidium corneum*	+	0	0	0.03	1		*Bryopsis muscosa*		+	0	0	0.03	1
*Pterocladiella capillacea*	+	0	0	0.03	1		*Bryopsis plumosa*		+	0	0	0.03	1
*Chondracanthus acicularis*	1	1	+	1.70	3		*Bryopsis secunda*		+	+	0	0.07	2
*Chondracanthus teedei*	0	1	+	0.87	2		*Caulerpa prolifera*		1	+	1	1.70	3
*Rissoella verruculosa*	+	+	0	0.07	2		*Codium bursa*		0	1	1	1.67	2
*Gracilaria bursa-pasoris*	1	+	+	0.90	3		*Codium effusum*		1	+	1	1.70	3
*Gracilariopsis longissima*	+	+	0	0.07	2		*Codium fragile*		1	0	1	1.67	2
*Hypnea musciformis*	2	2	1	10.83	3		*Codium tomentosum*		0	1	1	1.67	2
*Grateloupia filicina*	+	0	+	0.07	2		*Chaetomorpha aerea*		1	1	2	6.67	3
*Peyssonnelia polymorpha*	+	0	+	0.07	2		*Chaetomorpha mediterranea*		1	1	1	2.50	3
*Peyssonnelia rubra*	0	0	+	0.03	1		*Cladophora coelothrix*		0	1	+	0.87	2
*Peyssonnelia squamaria*	+	0	0	0.03	1		*Cladophora laetevirens*		1	1	1	2.50	3
*Sphaerococcus coronopifolius*	0	+	0	0.03	1		*Cladophora prolifera*		+	0	0	0.03	1
*Nemalion helminthoides*	+	+	0	0.07	2		*Acetabularia acetabulum*		+	0	0	0.03	1
*Liagora viscida*	+	0	1	0.87	2		*Bldingia marginata*		+	+	+	0.10	3
*Gastroclonium clavatum*	+	0	0	0.03	1		*Ulva compressa*		1	1	1	2.50	3
*Irvinea boergesenii*	+	0	0	0.03	1		*Ulva intestinalis*		+	0	0	0.03	1
*Cutleria adspersa*	0	0	+	0.03	1		*Ulva linza*		+	+	0	0.07	2
*Dictyota dichotoma*	1	1	0	1.67	2		*Ulva prolifera*		+	1	1	1.70	3
*Dictyota fasciola*	1	+	0	0.87	2		*Ulva clathrata*		2	1	1	6.67	3
*Dictyopteris polypodioides*	2	0	1	5.83	2		*Ulva fasciata*		+	+	+	0.10	3
*Dictyota spiralis*	0	+	1	0.87	2		*Ulva lactuca*		1	1	1	2.50	3
*Padina pavonica*	0	0	+	0.03	1		*Ulva rigida*		2	1	1	6.67	3
*Asperococcus bullosus*	+	0	+	0.07	2								
							Number of species per statement		89	68	72	130.4	
							Average number of species per statement		76.33			Pr: the presence of the species	
							R/P per statement		1.61	1.45	1.07		
							R/P Average		1.37				
							Diversity index H’		5.56				
							Evenness E		0.82				

**Table 4.** Analytical parameters at the Small Port Station.

	∑RMG	Dri	Q̄	DQ̄
Chlorophyceae	31.69%	24.30%	19	25.11%
Phaeophyceae	42.61%	32.67%	24	31.72%
Rhodophyceae	56.10%	43.02%	32.66	43.17%
Total	130.40%			

**Table 5.** Taxonomic diversity indices.

Region	S	delta	delta*	delta+
Small port	109	79.98	81.80	83.99
Salamandre	57	72.163	76.26	77.84
Expected		160.61	78.59	83.37
